# Supplementary figures and images for: A Two-tiered functional screen identifies herpesviral transcriptional modifiers and their essential domains
Source: PLoS Pathog. 2022 Jan 18;18(1):e1010236. doi: 10.1371/journal.ppat.1010236 (PMC8797222; doi:10.1371/journal.ppat.1010236)

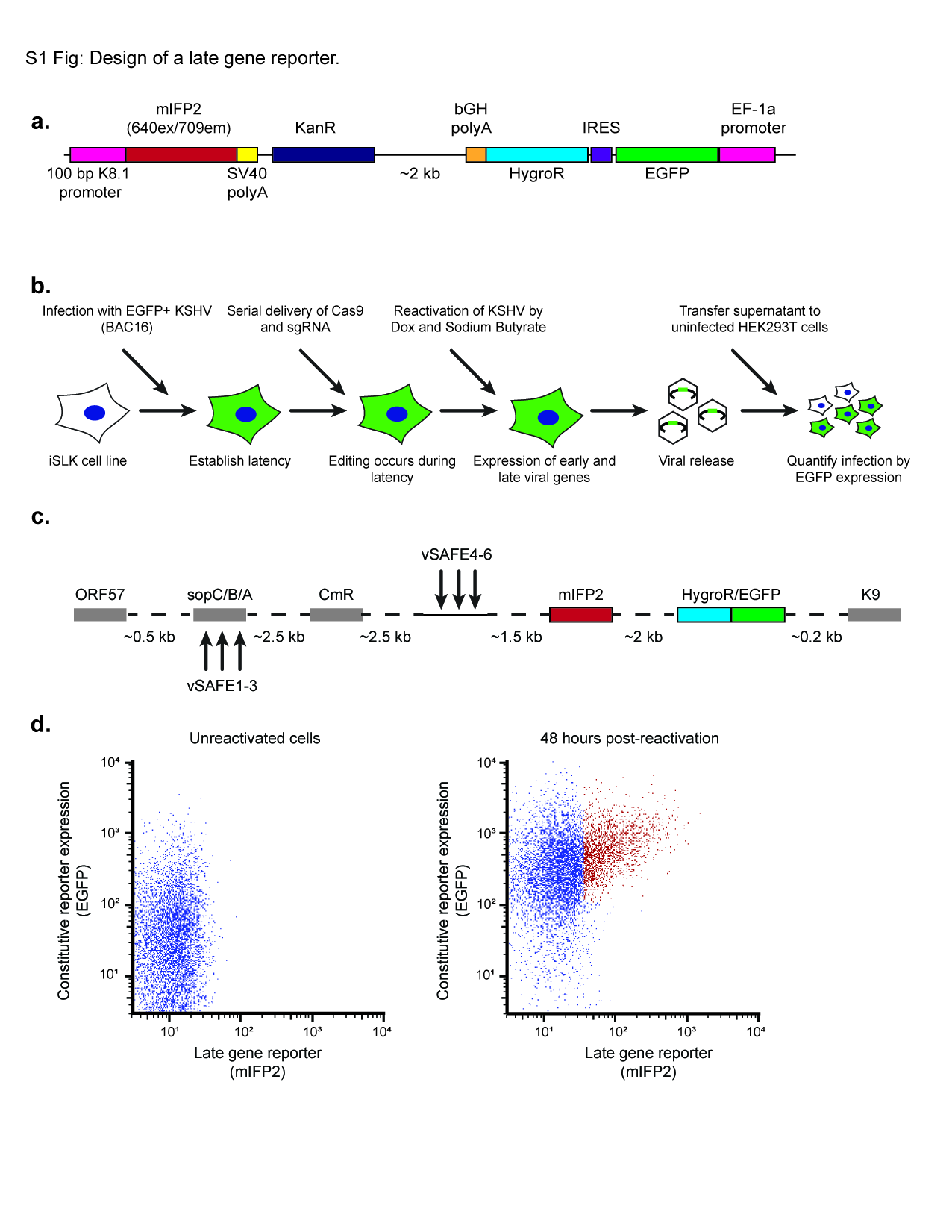

Supplement: S1 Fig — a) Design of a late gene reporter. A cassette expressing the far-red fluorescent protein driven by the 100 basepair promoter sequence of the KSHV late gene K8.1 was inserted upstream of the EGFP cassette of the BAC16 KSHV genome. A KanR cassette was included to allow selection in bacteria. b) Delivery and editing during latent infection of iSLK cells. Cas9-blast was lentivirally delivered to BAC16 infected iSLK lines. After selection, mU6-driven sgRNAs were delivered lentivirally. Cells were maintained in a latent state for 1–2 weeks to allow sufficient time for editing before reactivation by doxycycline and sodium butyrate. c) Schematic indicating targeting of vSAFE sgRNAs. d) Example flow data showing reactivation and expression of late gene reporter cells. Blue cells indicate late gene reporter negative cells, and red cells indicated late gene reporter positive cells. iSLK cells infected with the K8.1pr-mIFP2 BAC16 virus were reactivated with doxycycline and sodium butyrate, then fixed and analyzed 48 hours later by flow cytometry. EGFP is activated at 488 nm with a 595 LP, 525/50 filter. mIFP2 is activated at 640nm with a 750LP, 780/60 filter. (TIF) [file ppat.1010236.s001.tif]

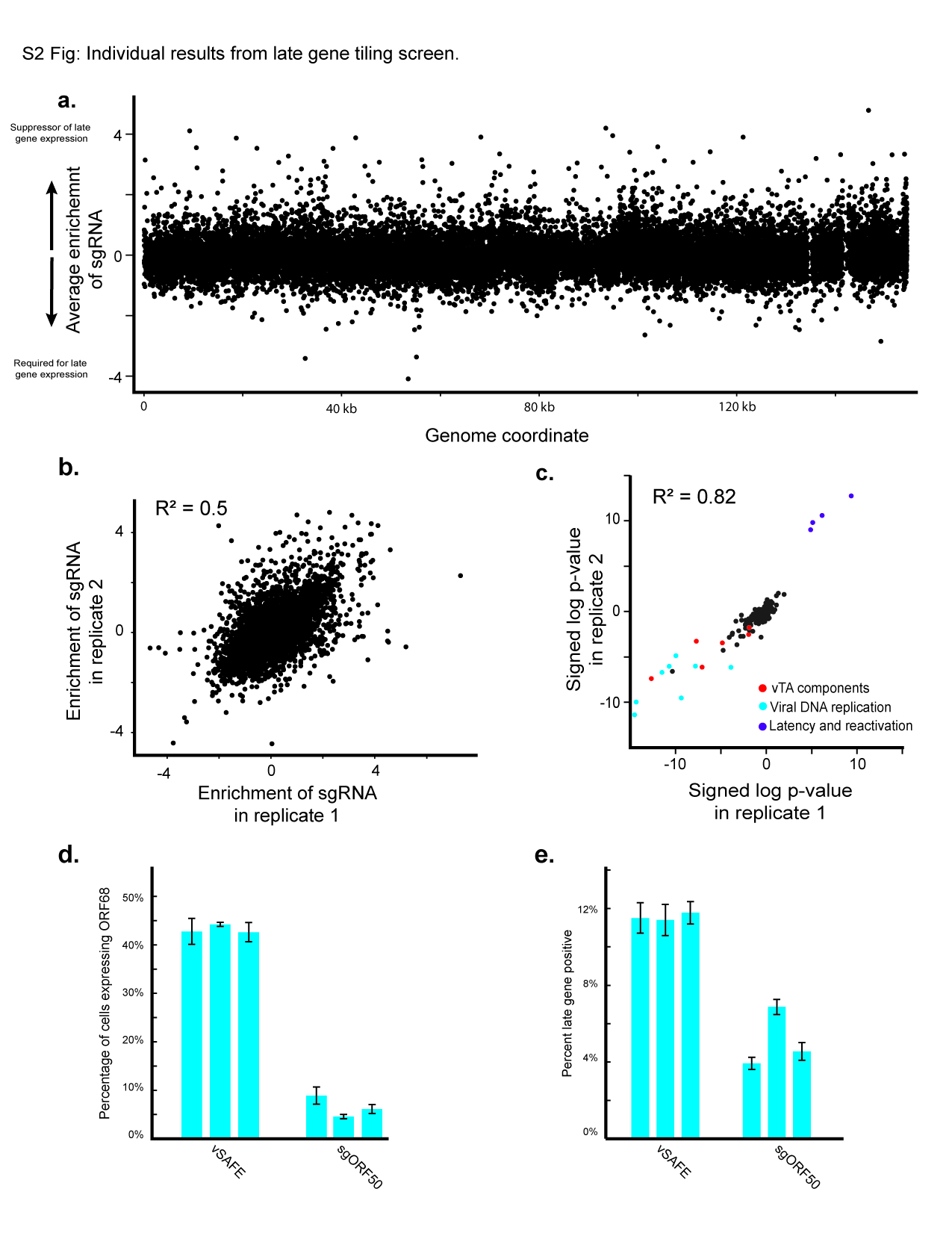

Supplement: S2 Fig — a) Average enrichment score from two replicates across the 154 kb genome. b) Guide-level reproducibility of sgRNA enrichments from two independent replicates (R2 = 0.5; p<10−200). Positive value indicates the sgRNA is enriched in the late-gene expressing fraction and thus promotes late-gene expression; negative value indicates the sgRNA is depleted from the late-gene expressing fraction and thus suppresses late gene expression. c) Gene-level reproducibility of p-values from two replicates (R2 = 0.82; p<10−70). To calculate significance, sgRNAs targeting each annotated region of the genome were grouped and a signed log Mann-Whitney p-value was calculated comparing each viral region to the negative control sgRNAs. A positive log p-value indicates this region promotes late gene expression when disrupted, and a negative log p-value indicates this region is required for expression of late genes. d) Reactivation and early gene expression of cells containing ORF50-targeting guides was measured using a KSHV virus containing a HaloTag fusion to a viral early gene, ORF68. Error bars are standard error from four independent reactivations. e) Expression of late gene reporter in cells containing ORF50-targeting guides. Error bars are standard error from four independent reactivations. (TIF) [file ppat.1010236.s002.tif]

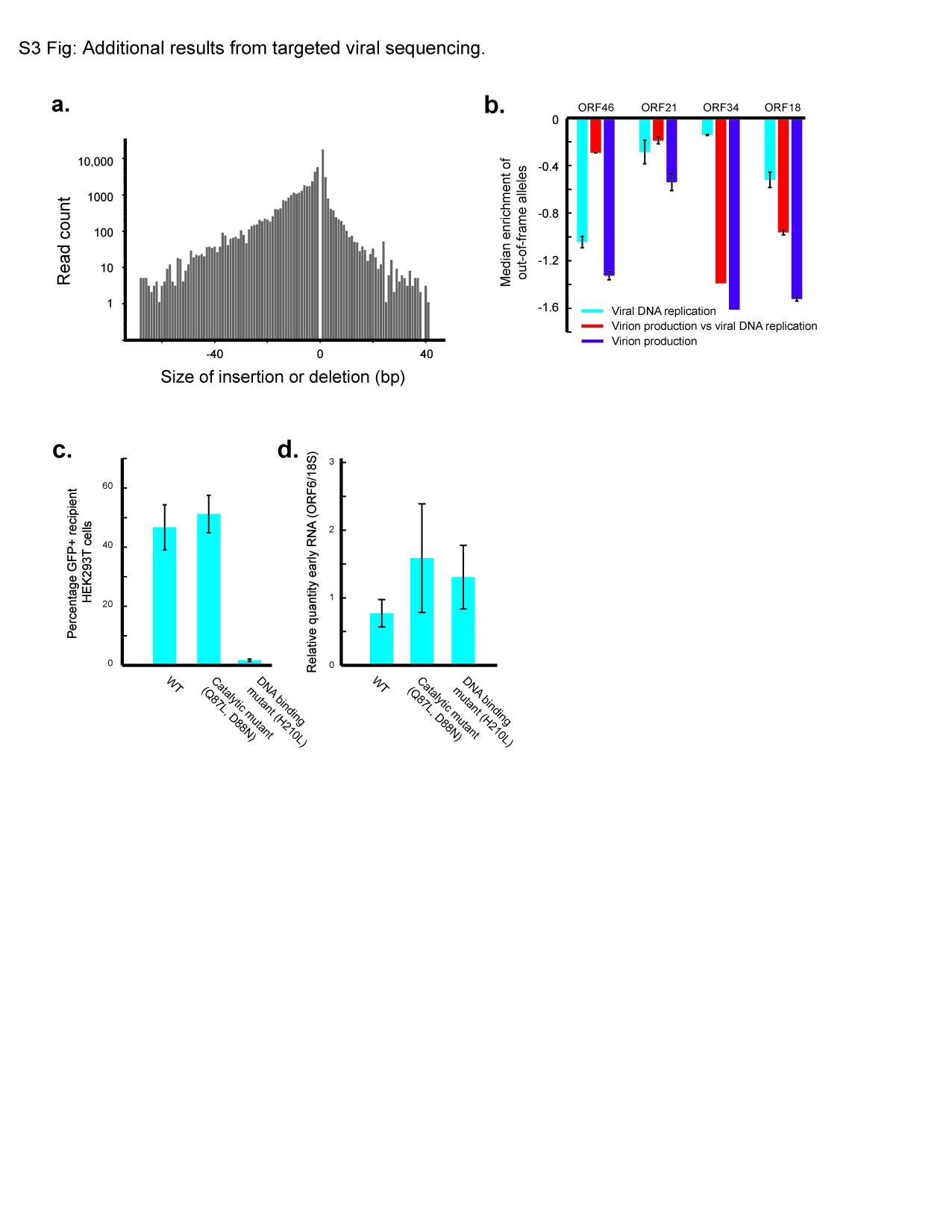

Supplement: S3 Fig — a) Representative spectra of indel sizes in a single replicate of latent genome. b) Median enrichment across the coding region of the gene of out-of-frame indels in replicating cells relative to latent cells (Viral DNA replication), the supernatant relative to latent cells (Virion production), or supernatant relative to replicating cells (Viral DNA replication vs Virion production). Error bars are standard error from two replicates. One replicate of ORF34 supernatant sample was excluded due to uneven coverage. c) Virion production was measured for iSLK cells infected with KSHV encoding either wildtype ORF46, a catalytic domain mutant of ORF46 (Q87L, D88N), or a DNA-binding domain mutant of ORF46 (H210L). Cells were reactivated, and after 72 hours supernatant was filtered and transferred to uninfected HEK293T cells. Infection was monitored by expression of the BAC16-encoded, constitutive EGFP. Error bars are standard error from three technical replicates. d) RNA expression of a viral early gene ORF6. 24 hours after reactivation, RNA was extracted, and RT-qPCR was performed with primers targeting the coding region of the viral early gene ORF6. Primers targeting the host 18S RNA were used as a control. Error bars are standard error from four technical replicates. (TIF) [file ppat.1010236.s003.tif]
